# Targeting the crosstalk between Alzheimer’s disease and gastrointestinal cancers

**DOI:** 10.1186/s10020-026-01545-x

**Published:** 2026-06-30

**Authors:** Rui Gong, Hao Wang, Lei Cao, Haijing Niu, Axel Rominger, Zhongguang Luo, Ruiqing Ni

**Affiliations:** 1 Department of Digestive Disease, Huashan Hospital, Fudan University, Shanghai, 200040 China; 2 Hepatobiliary Surgery, Department of General Surgery, Huashan Hospital & Cancer Metastasis Institute, Fudan University, Shanghai, 200040 China; 3 Department of Nuclear Medicine, Inselspital, Bern University Hospital, University of Bern, Bern, 3010 Switzerland; 4 State Key Laboratory of Cognitive Neuroscience and Learning &IDG/McGovern Institute for Brain Research, Beijing Normal University, Beijing, 100875 China; 5 Department of Neurobiology, Care Sciences and Society, Center for Alzheimer Research, Karolinska Institutet, Stockholm, Sweden

**Keywords:** Alzheimer's disease, Gastrointestinal cancer, Gut‒brain axis, Pancreatic cancer, Pancreas‒brain axis, Organ‒organ communication

## Abstract

Epidemiological studies have revealed an inverse association between Alzheimer's disease and cancer. Here, we discuss the mechanisms involved in the relationship between Alzheimer's disease and gastrointestinal cancers, particularly pancreatic and gastric-colorectal cancers. The gut‒brain axis and pancreas‒brain axis connect the central nervous system with peripheral organs and form immune‒metabolic networks. We focus on bidirectional tumor–brain communication, involving cell-death pathways, apoptosis, metabolic dysregulation, microbiota, metabolic dysregulation, neuroinflammation, immune system and sensory–sympathetic circuits, and neural remodeling. Furthermore, we discuss potential integrated, multitarget therapeutic strategies, including metabolic regulation, microbiome interventions, and immune modulation. Prospective longitudinal cohorts incorporating prediagnostic exposures and molecular pathology are needed to establish temporality.

## Introduction

Alzheimer's disease (AD) and cancer are two of the most prevalent diseases, collectively affecting more than 100 million people worldwide. AD is characterized by progressive cognitive decline and is pathologically characterized by the aberrant accumulation of amyloid-β (Aβ) plaques and neurofibrillary tau tangles, leading to neurodegeneration. Cancer is characterized by aberrantly acquired functional capabilities (hallmarks), including the ability to sustain proliferative signaling, inactivate growth suppressors, resist programmed cell death, regulate cellular metabolism, evade immune destruction, and activate invasion and metastasis (Hanahan 2026). The hallmarks of cancer include its phenotypic characteristics, ability of cells to populate cancer microenvironments, and systemic interactions (Hanahan 2026). Despite the fundamental cellular differences between these disorders, epidemiological research has indicated a significant inverse relationship (Table 1): individuals with AD have a lower risk (25–61%) of developing cancer (Driver et al. 2012; Ma et al. 2025), and in turn, a cancer diagnosis is associated with an 8–35% reduction in AD incidence, depending on the cancer type, population, and analytic method (Ma et al. 2025; Ospina-Romero et al. 2020). The risk of AD is lowest among survivors of smoking-related cancers and is not primarily explained by survival bias (Driver et al. 2012). Moreover, several pathological studies revealed that a cancer diagnosis was associated with a lower burden of AD pathologies and less cognitive impairment and that a cancer diagnosis directly affected the patient’s cognitive score and indirectly affected the patient’s condition via AD pathologies (Teipel et al. 2025; Karanth et al. 2022). Furthermore, genetic variants that increase the risk of sporadic AD, such as apolipoprotein E ε4, have been shown to be associated with tumor- suppressive activity in cancers (Barakji et al. 2025). Similarly, genes involved in protein folding, DNA repair, and cell-cycle control can have opposite associations with cancer versus neurodegeneration (Winkler et al. 2023). Body–central nervous system communication has emerged as a critical bidirectional regulator of physiological homeostasis and has complex regulatory functions in pathological states. It involves multiple systems, including the autonomic nervous system (sympathetic and parasympathetic), enteric nervous system, hypothalamic‒pituitary‒adrenal axis, gut microbiota and their metabolites, enteroendocrine cell-derived hormones and neurotransmitters, immune system, and inflammation. Recent evidence has suggested that specific molecular or cellular mechanisms confer “protection” to tumors, with survival and biological effects against neurodegeneration (Li et al. 2026). Additionally, bilateral body–brain communication could restrain cancer immunity via a sensory sympathetic axis, as demonstrated in a lung adenocarcinoma model (Wei et al. 2026).

**Table 1:** Key epidemiological studies reporting an inverse association between Alzheimer's disease and cancer

Cancer type	Study population (cohort)	Sample size	Age (yrs)	F%	HR/OR/RR/SIR for AD	Risk reduction%	Ref
All	Framingham Heart Study	1278	~ 76	61	HR: 0.6 (0.47–0.97) Smoking related cancers HR: 0.26 (0.08–0.82); non-smoking related cancers (0.82, 0.57 to 1.19)	33	Driver et al. 2012)
All (meta-analysis of 22 studies)	Pooled (multiple cohorts)	Variable (meta-analysis), 9,630,435	65–85	55–60	HR: 0.75 (0.61–0.93)	11	Ospina-Romero et al. 2020)
All	Danish national registry cohort	949,309	67	52	SIR: 0.94 (0.92–0.96)	6	Ording et al. 2020)
All	Autopsy cohort	1503	~ 86	58	OR 0.62 (0.45–0.85) for AD neuropathology	38	Karanth et al. 2022)
All	UK biobank	263,151	60	54	HR: 0.85 (0.74–0.98); male genital cancer 0.53 (0.29–0.97)	15	Zhang et al. 2022)
All	Korean NHIS cohort	123,320	> 70 (over 75%)	57	HR: 0.63 (0.59–0.68), pancreatic cancer HR: 0.50, hepatic cancer HR: 0.60, gastric cancer HR: 0.63, kidney cancer HR: 0.63, lung cancer HR: 0.64, thyroid cancer HR: 0.65, colorectal cancer HR: 0.67, gallbladder and biliary duct cancer HR: 0.73, hematologic malignancy cancer HR: 0.73, and bladder cancers HR: 0.76	37	Kang et al. 2023)
All	UK CPRD cohort	3,308,745	66	50	HR: 0.75 (0.74–0.76), Breast cancerHR: 0.82 (0.80–0.85) Prostate cancer HR: 0.68 (0.66–0.70) Lung cancerHR: 0.71 (0.65–0.76) Colorectal cancer HR:0.72 (0.70–0.75) Melanoma cancer HR:0.79 (0.75–0.83)	25	Bassil et al. 2024)
All (meta-analysis of 42 studies)	Pooled (multiple cohorts)	Variable (meta-analysis) 3,615,269	> 50	/	RR 0.88 (0.78‐0.99); lung cancer RR: 0.83 (0.73–0.95); colorectal cancer RR: 0.87 (0.80–0.95); non‐melanoma skin cancers RR: 0.95 (0.91‐0.99)	12	Ma et al. 2025)
All	NACC	2288	77	48	OR: for neuritic plaques ~ 0.9; Braak stage ~ 0.85		Teipel et al. 2025)

*F%* Female%, *HR/OR* 95% CI for AD risk in cancer patients (or vice versa), *AD* Alzheimer's disease, *CI* Confidence interval, *HR* Hazard ratio, *OR* Odds ratio, *RR* Relative risk, *SIR* Standardized incidence rate ratios

**Fig. 1:**
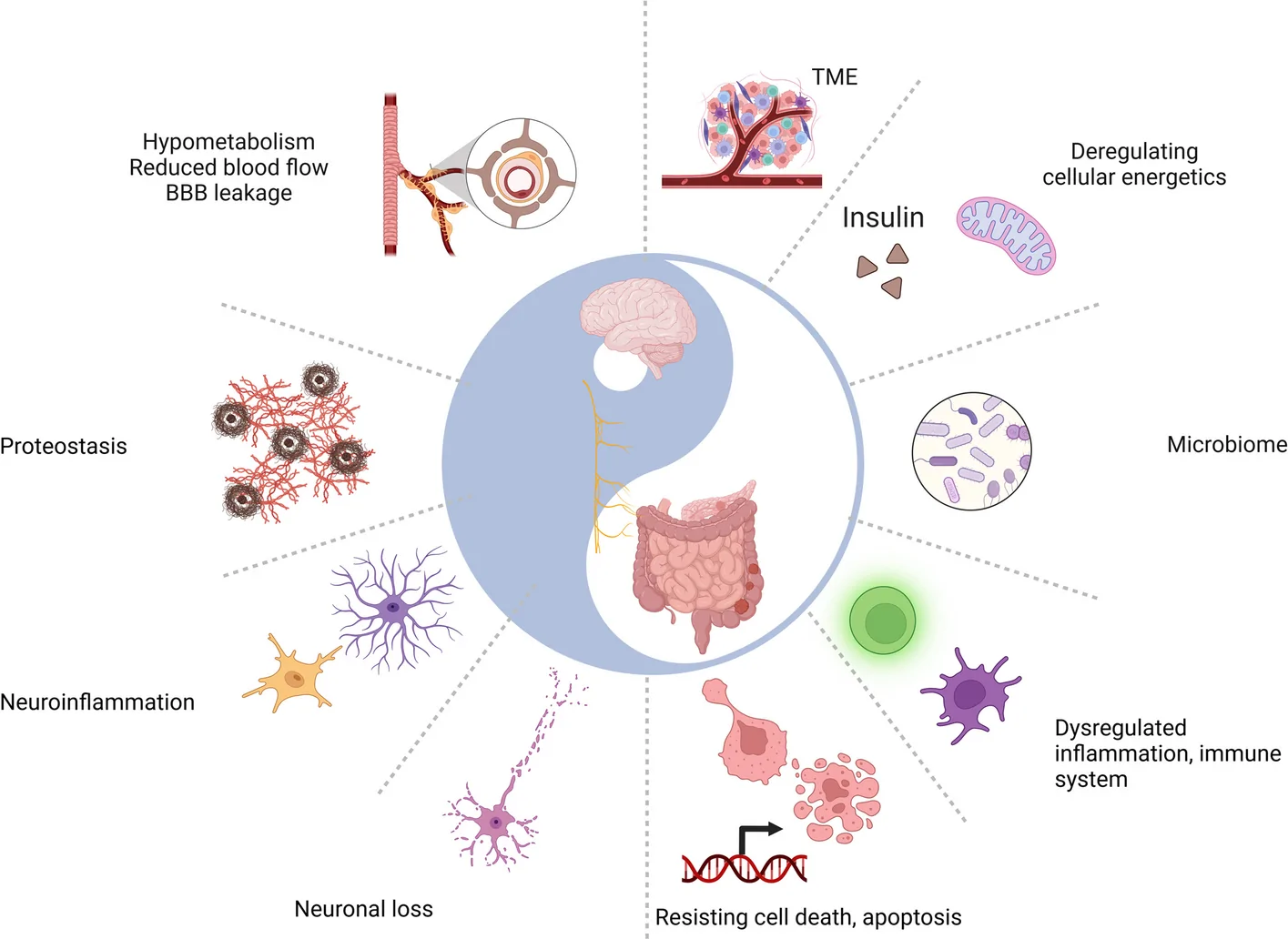
Opposing regulation of shared pathways in Alzheimer’s disease and gastrointestinal cancer. AD involves progressive synaptic loss, neuronal death, hypometabolism, reduced cerebral blood flow, blood–brain barrier (BBB) leakage, protein clearance failure, proteostasis, and chronic neuroinflammation, leading to neurodegeneration. In contrast, GI cancer cells exhibit uncontrolled proliferation, survival, and invasion; hypermetabolism; enhanced insulin and growth factor signaling; immune suppression; increased cellular energetics; angiogenesis; resistance to apoptosis; and alterations in the gut microbiome and tumor microenvironment (TME). Figure created in biorender.com. https://BioRender.com/oque0wr

Gastrointestinal (GI) cancers account for more than 25% of global cancer incidence and 30% of cancer-related deaths (Li et al. 2025). Although some GI cancers are declining in age-standardized rates, the incidence of pancreatic cancer is increasing, resulting in a greater burden of GI malignancies (Li et al. 2025). The negative association between GI cancers and AD has been shown to be a 15–30% reduction in AD risk (Ospina-Romero et al. 2020). The GI tract is the body’s largest immune and endocrine organ and is important for shaping the neural and tumor microenvironments (TMEs). The observed inverse relationship between AD and cancer suggests a fundamental imbalance that can be framed within a yin-yang model: AD corresponds to a loss-of-function, yin-deficient state characterized by hypometabolism and neurodegeneration. In contrast, cancer represents a gain-of-function, yang-excess state characterized by hypermetabolism and proliferation (Fig. 1). Many cellular pathways that control the cell cycle, apoptosis, immune responses, inflammation, and metabolism are shared between cancer and AD but are dysregulated in opposite directions, creating a trade-off between cancer risk and neurodegeneration risk. However, the current evidence for this inverse association is largely derived from cross-sectional, case‒control, and case-based studies, which cannot establish causality or account for the decades-long prodromal phases of both diseases. Prospective longitudinal cohort designs are essential for disentangling the temporal dynamics of this relationship.

### Balance of the cell cycle and signaling

In cancer, increased heat shock protein (Hsp) and proteasome activity promotes oncoprotein stabilization and enables tumor cells to adapt to proteotoxic stress (Wang et al. 2026). Cancer cells evade cell cycle checkpoints and proliferate continuously as damaged cells continue dividing (Hanahan 2026). Pathways such as the p53-retinoblastoma protein pathway, wingless-related integration site (Wnt)/β-catenin (Liu et al. 2022), autophagy–lysosomal pathway (Lee et al. 2022), protein phosphatase 2, prolyl-isomerase Pin1 and microRNAs (e.g., miR-21 and miR-29) are opposingly regulated in AD and cancer. Protein quality control systems, the ubiquitin‒proteasome system, and the autophagy‒lysosomal pathway are compromised in AD. Earlier studies have shown that molecular chaperones (Hsp70 and 90) and multichaperone complexes are critical regulators of tau in AD (Moll et al. 2022). This contributes to the impaired clearance and aberrant accumulation of Aβ and tau aggregates. Pin1 regulates postphosphorylation protein folding. Reduced Pin1 expression in AD has been shown to lead to tau hyperphosphorylation and neurofibrillary tangle formation (Lim et al. 2008), whereas Pin1 overexpression in cancer promotes the stabilization of oncoproteins (cyclin D1, β-catenin, and c-Myc) and tumor progression (Lu and Hunter 2014). In addition, reduced Wnt signaling in AD has been shown to increase Aβ production, tau hyperphosphorylation and synaptic loss, whereas in cancer, mutations inWnt pathway genes (APC, CTNNB1 or RNF43) lead to β-catenin nuclear accumulation and MYC and CCND1 activation (Liu et al. 2022). In addition, glycogen synthase kinase-3 beta (GSK-3β) plays an important role in AD: overactivated GSK-3β leads to downstream PI3K/Akt signaling dysfunction, tau hyperphosphorylation and increased Aβ production via amyloid precursor protein processing. In cancer, GSK-3β activity is context dependent across tumor type, stage, and subcellular localization. It can act as a tumor suppressor by inhibiting Wnt/β-catenin signaling and promoting apoptosis. However, in many GI cancers, GSK-3β is inactivated, which relieves the suppression of β-catenin activity and is associated with increased proliferation, chemoresistance, and immunosuppression, including stabilization of PD-L1 expression (Lauretti et al. 2020). In AD, mature neurons aberrantly re-enter the cell cycle and show increased activation of cell- death pathways. p53 activation contributes to neuronal apoptosis and mitochondrial dysfunction. Owing to their abnormal checkpoint function, these neurons cannot complete division and instead undergo apoptosis, resulting in neuronal loss. In cancer, p53 mutation or MDM2 overexpression impairs p53-mediated apoptosis and promotes uncontrolled proliferation (Wade et al. 2013). These molecular 'toggle switches' may change toward enhanced tumor- suppression but increase the vulnerability to neurodegeneration or vice versa.

### Inflammation, immune dysregulation, and tumor-promoting inflammation

Cancer often involves “turning down” immune surveillance, where proinflammatory factors promote tumor growth while suppressing antitumor immunity (Hanahan et al. 2025; Visser and Joyce 2023). The GI TME is highly dynamic and immunosuppressive. These include insufficient effector T-cell infiltration, enrichment of regulatory T cells and myeloid-derived suppressor cells, M2 polarization of tumor-associated macrophages, and imbalanced cytokine networks. a complex immune evasion network. A complex network facilitating immune evasion is established through the secretion of various molecules by both tumor cells and stromal cells. Key components include immune checkpoint molecules (programmed death-ligand 1 (PD-L1), cytotoxic T-lymphocyte associated protein 4 ligands), immunosuppressive factors (transforming growth factor-β, Interleukin 10, and vascular endothelial growth factor), and chemokines (chemokine ligand 2 and CXC motif chemokine 12).

In AD, chronic neuroinflammation and dysregulated innate immunity contribute to Aβ and tau pathology and neurodegeneration (Maschio et al. 2025). This may diminish the body's surveillance and clearance capacity for other pathologies, including neurodegeneration. A recent study revealed that tumor-secreted cystatin-C can traverse the blood‒brain barrier in AD mice and activate microglial triggering receptors expressed on myeloid cell 2 receptors, thereby promoting Aβ plaque clearance (Li et al. 2026). Using RNA and T-cell receptor sequencing techniques, a recent postmortem study revealed that clonal CD8^+^ T cells populate leptomeninges and form regulatory networks to control immune cells in the AD brain (Hobson et al. 2026). Fibrillar Aβ-targeting CD4^+^ T cells (Boskovic et al. 2026) and astrocytes (Chen et al. 2026) were engineered with chimeric antigen receptors to reduce Aβ pathology and improve cognition in AD mice. An earlier study revealed a correlation between changes in PD-1/PD-L1 expression on peripheral T-cell subsets and AD disease stage (Wu et al. 2022). Preclinical and clinical meta-analyses have reported the potential beneficial effects of PD-1/PD-L1 blockade in AD (Yoon et al. 2026) and the anti-PD-L1 antibody IBC-Ab002 in people with early AD. However, further experimental studies are needed to elucidate the mechanism involved in influencing Aβ or tau pathology in AD.

Additionally, peripheral organ inflammation, such as inflammatory bowel disease, gut dysbiosis, chronic pancreatitis, and type 2 diabetes-associated inflammation, can transmit proinflammatory signals to the central nervous system (Zhang et al. 2022; Kong et al. 2024). AD patients exhibit altered gut microbiome composition, including reduced microbial diversity, depletion of beneficial bacteria (e.g., *Bifidobacterium* and *Lactobacillus*), and increased abundance of proinflammatory bacteria (e.g., *Escherichia* and *Enterobacteriaceae*). Preclinical studies have demonstrated that *Bifidobacterium breve* and *Lactobacillus plantarum* can reduce Aβ deposition in the brains of AD mice (Abdelhamid et al. 2022). Dysbiosis has been shown to compromise intestinal barrier integrity and allows bacterial lipopolysaccharides, peptidoglycans, and other pathogen-associated molecular patterns to enter systemic circulation.

Two key pathways involved in microbiome–brain communication have been identified. First, short-chain fatty acids (SCFAs), such as butyrate, acetate, and propionate, are produced by beneficial bacteria via dietary fiber fermentation. SCFAs strengthen intestinal barrier integrity by upregulating the expression of tight junction proteins (claudin-5, occludin, and ZO-1) and cross the blood‒brain barrier via monocarboxylate transporters. SCFAs modulate microglial activation by inhibiting the activity of histone deacetylases, reducing the production of proinflammatory cytokines (tumor necrosis factor-alpha, interleukin-1β, and interleukin-6), and promoting a neuroprotective, phagocytic microglial phenotype (Silva et al. 2020). In addition, gut dysbiosis increases intestinal permeability, allowing lipopolysaccharide and other pathogen-associated molecular patterns (e.g., peptidoglycans and flagellin) to enter the systemic circulation. Bacterial lipopolysaccharide binds to Toll-like receptor 4 on peripheral monocytes and macrophages, activating nuclear factor kappa-light-chain-enhancer of activated B-cell signaling and inducing systemic production of proinflammatory cytokines. These cytokines reach the brain via active transport across the blood‒brain barrier, binding to receptors on brain endothelial cells, triggering local cytokine production, and activating vagus nerve afferents, which transmit inflammatory signals to brainstem nuclei. In the central nervous system, these cytokines further activate microglia and astrocytes in the brain, leading to increased reactive oxygen species and nitric oxide production, increased Aβ generation and tau phosphorylation, and decreased Aβ clearance and synaptic plasticity (Chen et al. 2023).

### Metabolic dysfunction and the gut microbiome

The pancreas is unique because it is both a digestive organ that secretes digestive enzymes and an endocrine organ that secretes insulin, glucagon, and pancreatic polypeptides, which are critical for energy metabolism and neurological function. Dysbiosis of the gut microbiome influences the secretion of incretin (such as glucagon-like peptide-1), subsequently affecting pancreatic islet function and systemic metabolism. In addition, the pancreatic secretions of digestive enzymes and bicarbonate modulate the intestinal pH and microbial ecology. An earlier study demonstrated that a microbially responsive subset of viscerofugal cocaine- and amphetamine-regulated transcript^+^ neurons enriched in the ileum and colon can modulate feeding and glucose metabolism (Muller et al. 2020). In addition, in cachexia in advanced pancreatic cancer, circulating monocytes and macrophages pass through the blood‒brain barrier to infiltrate the hypothalamus and secrete proinflammatory cytokines that interfere with hypothalamic appetite regulatory circuits (Burfeind et al. 2020). This leads to the suppression of agouti-related peptide/neuropeptide Y neuronal activity while activating proopiomelanocortin neurons, resulting in appetite suppression and increased energy expenditure (Solis et al. 2024).

AD is considered “type III diabetes” (Kellar and Craft 2020), with insulin resistance as a core metabolic feature. AD patient brains exhibit reduced insulin receptor signaling pathway activity, abnormal insulin receptor substrate-1 phosphorylation patterns and downstream signaling disruption. This leads to mitochondrial dysfunction, reduced adenosine triphosphate generation, and metabolic dysfunction with impaired neuronal glucose uptake. Peripheral hyperinsulinemia saturates insulin-degrading enzymes that normally degrade insulin and Aβ, can lead to compromised Aβ clearance, and can impair cerebrovascular endothelial function and the blood‒brain barrier. In addition, the resulting glycogen synthase kinase-3β overactivation can cause tau hyperphosphorylation.

### Sensory–sympathetic circuit, neural remodeling

Cancer cells express synaptic-associated proteins and neurotransmitter receptors and can form synapse-like connections with neural axons for direct neural signal regulation. Neuron‒tumor bidirectional interactions can drive tumor proliferation, invasion and metastasis through neurotransmitter-activated signaling pathways and epithelial‒mesenchymal transition. In addition, it affects chemoresistance, antiapoptotic pathways, and immunosuppression through the recruitment of tumor-associated macrophages and myeloid-derived suppressor cells by neuropeptides and neurotransmitters. For example, in colorectal cancer, elevated neuronal activity in the septal region is correlated with larger primary tumors, and increased levels of the neurotransmitter γ-aminobutyric acid (GABA) can predict poor prognosis. Activation of the septoenteric circuit by cancer cells induces the release of GABA from enteric cholinergic neurons, which also activates GABA_A_ receptors on tumor cells to sustain tumor growth (Li et al. 2025; Huang et al. 2022). In addition, activation of the GABA_B_ receptor inhibits glycogen synthase kinase-3β activity and CD8^+^ T-cell intratumoral infiltration and enhances β-catenin signaling and MC38 colon tumor cell proliferation (Huang et al. 2022). Multiomic screening revealed that MC38 colon cancer cells can release key cytokines, such as leukemia inhibitory factor and galectin-3, to trigger brain activation (Xu et al. 2024) and that pharmacologic or genetic blockade of these signaling pathways can abolish brain responses and strongly inhibit tumor growth. Additionally, pancreatic cancer cells remodel the neural microenvironment by secreting neurotrophic factors, such as nerve growth factor and brain-derived neurotrophic factor, to promote axonogenesis (Jiang et al. 2025; Thiel et al. 2025). Nerve growth factor also promotes gastric tumorigenesis through aberrant cholinergic signaling (Hayakawa et al. 2017). In turn, neurons release serine to support mRNA translation in pancreatic cancer (Banh et al. 2020).

### Therapeutic strategies and outlook

Recent insights into the shared mechanisms among cancer, neurodegeneration, and metabolic disease have opened up diverse therapeutic avenues (Ma et al. 2025). These strategies can be broadly categorized into targeting metabolic dysfunction, the microbiome, the inflammatory immune system and the sensory-to-sympathetic pathway.

Interventions targeting insulin resistance and metabolic dysregulation are at the forefront of therapeutic development for AD. The promising metabolism-targeted approaches being explored include intranasal insulin delivery, metformin, sodium‒glucose transport 2 inhibitors, and ketogenic diets (Corraliza-Gomez et al. 2025). While a preclinical study revealed that activation of 5' adenosine monophosphate-activated protein kinase by glucagon-like peptide-1 (GLP-1) receptor agonists mitigated pathology and cognitive impairment in AD mice (Zhang et al. 2025), the first two phase 3 clinical trials, EVOKE and EVOKE + that tested semaglutide in AD patients, reported negative results (Cummings et al. 2026). One possible reason is that these metabolism-suppressing agents (GLP-1 agonists) exacerbate “yin” and enhance the cerebral hypometabolism of AD.

Thyroid hormone replacement using levothyroxine could promote metabolism and countering the hypometabolic “yin” state in AD patients, although this has not yet been clinically tested. In the Framingham Study of 65,000 patients, abnormal thyroid function (low or high thyrotropin levels) was associated with increased AD risk in women, suggesting that thyroid hormone homeostasis is important for brain health during aging. Thyroid requirements often decrease with increasing age; therefore, an adaptive dosage is needed. While associations exist between thyroid levels and AD, a causal link between levothyroxine and AD has not been shown. Mixed results on the effect of thyroid hormone therapy on cognitive outcomes have been reported (Tan et al. 2008; Salehipour et al. 2023). Preclinical studies suggest that hypothyroidism can worsen AD-related pathology and microglial dysfunction, whereas T3/T4 replacement may improve neuronal metabolism and cognitive behavior and enhance Aβ clearance through improved glial and autophagic pathways (Kim et al. 2024). In addition to thyroid hormone, other metabolic promoters are being explored preclinically, such as thyroid hormone analogs (e.g., sobetirome), mitochondrial uncouplers (e.g., niclosamide) that increase adenosine triphosphate production efficiency, and ketone bodies (e.g., β-hydroxybutyrate from ketogenic diets or exogenous ketone esters) that provide alternative energy and inhibit histone deacetylases. Future studies are needed to evaluate the therapeutic window of metabolic promoters in AD, with parallel monitoring for neoplastic risk.

Additionally, because of the critical role of the gut microbiota in neurodegenerative diseases, the microbiome has been identified as a key therapeutic target for AD (Munir et al. 2024). Interventions such as probiotics, prebiotic supplementation, dietary interventions, and fecal microbiota transplantation are being tested to restore a healthy microbial balance. Specifically, beneficial strains such as *Lactobacillus* and *Bifidobacterium* can increase short-chain fatty acid production, reduce inflammation, and strengthen the intestinal barrier, all of which are correlated with improved cognitive performance. Furthermore, potential microglial-targeted therapies for AD, such as recombinant cystatin-C therapy, triggering receptor expression for myeloid cell 2 agonist development, screening of tumor secretomes, and immune checkpoints that may lower chronic brain inflammation in AD, are being explored (Li et al. 2026).

For cancer, therapies are being developed to directly modulate the influence of the nervous system. For example, in pancreatic ductal adenocarcinoma, where perineural invasion affects 80–100% of cases (Lovecek et al. 2025), disrupting the sensory-to-sympathetic pathway can enhance antitumour immune responses and inhibit metastasis (Wei et al. 2026). A previous preclinical study reported that stress-induced glucocorticoid surge and *Tsc22d3* upregulation can subvert therapy-induced anticancer immunosurveillance (Yang et al. 2019). Recent studies have shown the benefit of using psychological or pharmacological interventions to block adrenergic and inflammatory stress signaling, especially alongside cancer treatments (Eckerling et al. 2021). Furthermore, targeted approaches are emerging to manipulate specific neural signals, such as tumor-derived GABA (Huang et al. 2022), neuronal substance P (Padmanaban et al. 2024), or hypothalamic peptides such as α-melanocyte-stimulating hormone (Xu et al. 2022), to reverse immunosuppression and combat metastasis.

### Longitudinal cohorts with temporal information

Despite the progress in decoding the crosstalk underlying brain-body communication, a complete picture of this association is lacking (Ugai et al. 2025). A major limitation of the existing evidence is its reliance on cross-sectional, case‒controlled study designs. Given that both AD and GI cancers have lengthy prodromal phases that last decades, these designs cannot establish temporality or causality (Ogino et al. 2026). Neoplastic processes and AD pathologies (e.g., Aβ and tau accumulation) develop over many years. Prediagnostic factors, including lifelong environmental exposure, lifestyle (diet, physical activity, and smoking), chronic inflammation, and metabolic health, shape disease characteristics long before clinical presentation (Livingston et al. 2020). The high interindividual variability in these prediagnostic exposures may explain the heterogeneity observed in epidemiological studies. Therefore, prospective longitudinal cohort studies are critically needed to follow individuals from midlife through the development of both AD and cancer. This will allow understanding of the parallel/interacting trajectories over the life course and determine whether the inverse association reflects a true biological trade-off, shared risk factors with opposite effects, or residual confounding.

A promising methodological framework to address these gaps is the prospective cohort incident-tumor biobank method (PCIBM) (Ogino et al. 2026). This approach enables us to move beyond cross-sectional information and toward understanding the causal and temporal dynamics of the AD-GI cancer association. A recent study reported that long-term yogurt intake and the incidence of colorectal cancer were subclassified by the abundance of *Bifidobacterium* in tumors (Ugai et al. 2025). The PCIBM integrates long-term, prediagnostic exposure data (collected prospectively) with postdiagnostic tumor tissue molecular profiles (e.g., immune checkpoint expression and Wnt/β-catenin status) and AD-related biomarkers (e.g., Aβ, tau, and neuroinflammation). This enables the establishment of temporal sequences between prediagnostic exposures (e.g., metabolic syndrome, gut dysbiosis, chronic inflammation) and subsequent AD pathologies and GI cancer, taking into account the individual heterogeneity in disease trajectories.

Despite the progress in decoding the crosstalk underlying brain–body communication, a complete picture of this association is lacking (Swanton et al. 2024). Longitudinal cohort studies are needed to elucidate the causal relationship between AD and GI cancer. In addition, high-throughput omics technologies and single-cell analyses are needed to identify critical nodes and regulatory networks. Spatial transcriptomics and real-time imaging can reveal spatiotemporal dynamics. Moreover, the level of the neuroimmune–metabolic system should be integrated. Total-body positron emission tomography with, for example, [^18^F]fluorodeoxyglucose enables the measurement of impaired neuronal glucose uptake and simultaneously reveals how systemic pathology and organ function occur within a single, integrated dataset. Additional new tracers, such as those that trigger receptors expressed on myeloid cells 2, are helpful for visualizing alterations in the immune system.

Moving forward will require multidisciplinary integration, employing systems biology, network medicine, and precision medicine approaches. Given the complexity of these interconnected systems, a shift from single-target interventions toward integrated approaches might show greater promise.

## Data Availability

No datasets were generated or analysed during the current study.

## Abbreviations

Aβ: Amyloid-beta
AD: Alzheimer’s disease
GABA: γ-Aminobutyric acid
GI: Gastrointestinal
GLP-1: Glucagon-like peptide-1
Hsp: Heat shock protein
PD-1: Programmed death-1
PD-L1: Programmed death-ligand 1
PCIBM: Prospective cohort incident-tumor biobank method
Pin1: Peptidyl-prolyl cis–trans isomerase NIMA-interacting 1
TME: Tumor microenvironment
Wnt: Wingless-related integration site
